# How Is It Done?

**Published:** 1892-05

**Authors:** 


### HOW IS IT DONE?

Several months ago an itinerant mind reader exhibited in this place, and young Taylor attended his performance. Returning home, he playfully remarked to a young man who had accompanied him that he thought he would make a good mind reader, and that if the other would blindfold him and hide something he would find it for him. To have a little amusement he was duly blindfolded and told to find a book that had been hidden in an adjacent room. He grasped the hand of the young man who had hidden the book, but was utterly surprised to find that not only the book, but also its place of concealment, were impressed on his mind. He readily took the young man to the place were the book was and handed it to him. After this there were more or less frequent tests of his powers in finding things thus, while all hidden articles were always promptly located by him. Intermingled with these tests were others, such as willing him to do certain things. Say, for instance, that it was willed for him to take a particular flower of a number of flowers in a vase in the room, and to hand it to a certain young lady present; to remove the watch from the pocket of a certain gentleman and to put it into the pocket of another certain gentleman; to go to a library and take out some particular volume in it, and turn to a certain page and paragraph or sentence in it, and so on of other requests of this sort. All these were readily and accurately done by him, down to the minutest particular of the wish.

Any figure, or any number of figures, being thought of, he has readily announced what it or they were, calling them out singly or in combination as desired. Some time ago, knowing that he did not understand Latin, I improvised a short Latin sentence and asked him to tell me what it was. This was made out slowly, but quite accurately, the words being spelled out, letter by letter.

Upon what other grounds can we explain this telling of figures and calling out Latin than upon the silent impress of mind upon mind? This is the explanation, in fact, that young Taylor gives of his "mind reading." His great difficulty, he says, is to get a correct impression from some who either lack concentration of mind or allow the too frequent intrusion of other thoughts into it. For a good effect impressions must be forcible and sharp-cut, and the mind must be kept steadily and as exclusively as possible on the subject. He thinks the hand acts only as a conductor of impression, and regards it as indispensable for that purpose, as the current of impression is transmitted in this way, without which he could tell nothing. In conclusion, I may add that in his performances there is usually considerable disturbance of his physical being. His respiration often becomes slow and labored, pulse usually goes up from ten to twenty beats above normal to the minute, there is heavy sighing at times, and sometimes so much exhaustion as to necessitate temporary rest.

[The modest claim of young Taylor respecting his own share in the process of mind reading, points the way to a sublime truth. The mind reader is a mere instrument in the hands of invisible intelligences. He is simply and solely a medium, and the impressions he receives are wholly outside of himself. It is occultism, pure and simple, and every intelligent spiritualist knows it.—ED.]

